# Willingness to accept monkeypox vaccine and its correlates among men who have sex with men in Southern China: a web-based online cross-sectional study

**DOI:** 10.3389/fpubh.2024.1289918

**Published:** 2024-02-07

**Authors:** Xinju Huang, Zhifeng Lin, Jiao Qin, Dee Yu, Fei Zhang, Ganggang Fang, Xi Chen, Jinfeng He, Ping Cen, Mu Li, Rongjing Zhang, Tong Luo, Junjun Jiang, Sanqi An, Hao Liang, Li Ye, Bingyu Liang

**Affiliations:** ^1^Guangxi Key Laboratory of AIDS Prevention and Treatment, School of Public Health, Guangxi Medical University, Nanning, Guangxi, China; ^2^Kaiyuan Center for Disease Control and Prevention, Kaiyuan, Yunnan, China; ^3^Sanya Center for Disease Control and Prevention, Sanya, Hainan, China; ^4^Collaborative Innovation Centre of Regenerative Medicine and Medical BioResource Development and Application Co-constructed by the Province and Ministry, Life Science Institute, Guangxi Medical University, Nanning, Guangxi, China

**Keywords:** monkeypox (MPX), men who have sex with men (MSM), perception, vaccination, hesitancy

## Abstract

**Background:**

The May 2022 global outbreak of monkeypox (MPX) poses a threat to the health of men who have sex with men. However, there is limited data on the willingness of MSM to receive monkeypox vaccination in Southern China. This study aimed to assess the knowledge of MPX, concerns regarding MPX, and willingness to receive monkeypox vaccination, as well as their correlates, among MSM in China.

**Methods:**

We conducted a Web-based online survey of MSM in Southern China from August to September 2022. Data were collected on the socio-demographic characteristics, knowledge, worries, concerns regarding MPX and willingness to receive monkeypox vaccination. Multivariate logistic regression was employed to explore the factors associated with willingness to receive monkeypox vaccination.

**Results:**

A total of 1903 participants completed the survey. Among them, approximately 69.9% reported being aware of MPX awareness, 94.1% of the participants supported the promotion of monkeypox vaccination. The majority of participants (91.4%) expressed their willingness to receive monkeypox vaccination. Participants who considered monkeypox vaccination safe [adjusted odds ratio (aOR) = 4.82, 95% CI: 1.35–17.18], agreed on the necessity of government promotion of monkeypox vaccination in China (aOR = 6.03, 95% CI: 1.07–33.93), believed in prioritizing monkeypox vaccination for MSM (aOR = 5.01, 95% CI: 1.10–22.71), and had friends or sexual partners who had already received the monkeypox or smallpox vaccination (aOR = 10.37, 95% CI: 2.11–50.99) are more likely to be vaccinated. Conversely, married individuals (aOR = 0.13, 95% CI: 0.03–0.47), those engaging in anal sex 4–6 times per week in the past 3 months (aOR = 0.26, 95% CI: 0.09–0.77) expressed hesitancy toward monkeypox vaccination.

**Conclusion:**

There was a high willingness to receive monkeypox vaccination among MSM in China. The hesitancy toward the monkeypox vaccine can be effectively mitigated by addressing concerns about its safety and potential adverse reactions. Moreover, increasing acceptance of the monkeypox vaccination among MSM and their peers is crucial, as social influence significantly impacts vaccine attitudes and behaviors.

## Introduction

Since the outbreak of monkeypox (MPX) in May 2022, the pandemic has garnered widespread global concern (1). By October 2023, the World Health Organization (WHO) reported 91,788 cases and 167 deaths worldwide, with an average of approximately 752 cases per month (2). Despite WHO declaring on May 23, 2023, that the monkeypox epidemic does not constitute a “Public Health Emergency of International Concern” (PHEIC), MPX remains a significant global health threat.

Monkeypox virus (MPXV) is an enveloped, double-stranded DNA virus belonging to the Orthopoxvirus genus, and is a zoonotic virus (3). The clinical presentation of MPXV is similar to that of smallpox virus and is characterized by the presence of varicella or scabs on the skin, primarily affecting the trunk, face, and genital area (4, 5). The main modes of transmission include human-to-human transmission, respiratory transmission, and direct contact with the mucosa and skin of infected individuals. Currently, there are no specific treatments available for monkeypox, and only supportive care can be provided (6, 7).

The monkeypox outbreak, which has been ongoing since 2022, has seen a higher proportion of infections among men who are gay, bisexual or otherwise men who have sex with men (8–10). Furthermore, a majority of these individuals were found to be co-infected with human immunodeficiency virus (HIV) or other sexually transmitted infections (STIs) (11, 12). Studies have demonstrated that MPXV can replicate and be transmitted through semen (13), indicating that sexual transmission may be one of the routes of MPXV transmission (14, 15). The population of MSM is known to be at a high risk of HIV infection and other sexually transmitted diseases (STDs) and carries the highest burden of HIV infection in many countries (16). Therefore, focusing on the prevention of MPXV among the MSM population is of utmost importance.

Vaccination is one of the most cost-effective preventive methods for controlling infectious diseases. Currently, there are two main vaccines used to prevent monkeypox virus infection: ACAM2000 vaccine and JYNNEOS vaccine. The ACAM2000 vaccine, known as the smallpox vaccine, was licensed by the U.S. Food and Drug Administration (FDA) in August 2007. It is a live attenuated replicating vaccine approved under the Investigational New Drug application (IND) protocol for treatment of non-variola orthopoxvirus infections during outbreaks such as monkeypox. The JYNNEOS vaccine, also known as the Smallpox and monkeypox non-replicating live vaccine, was licensed by FDA in September 2019. It is a live attenuated, non-replicating vaccine for preventing smallpox and monkeypox in adults at high risk. These vaccines actively immunize against smallpox and are effective for monkeypox prophylaxis due to their cross-protection against related poxviruses (17–19). Given the rapid expansion of the MPX epidemic, the use of vaccines holds significant potential in effectively controlling its spread and impact.

Widespread concerns about vaccination, including uncertainties about safety, potential adverse reactions, and efficacy, can contribute to vaccine hesitancy and discourage individuals from getting vaccinated (20, 21). Therefore, the WHO declared vaccine hesitancy as one of the top 10 threats facing the world in 2019 (22). Similarly, factors such as doubts about the safety of the monkeypox vaccine, levels of worry about monkeypox, and negative perceptions related to COVID-19 vaccination were found to contribute to hesitancy toward receiving monkeypox vaccination (23, 24). Enhancing people’s knowledge about MPX and vaccines is therefore crucial in reducing vaccine hesitancy and improving the acceptance of monkeypox vaccines.

In addition to Europe and the United States, which have been severely affected by the MPX outbreak, there are currently many cases in Asia, including China. From January 1, 2022 to June 19, 2023, 160 cases of MPX infection have been reported in China, including mainland China (5), Hong Kong SAR (7), and Taibei (148) (2). While some surveys have examined the willingness to receive monkeypox vaccination in China (25–30), there is a lack of data regarding the willingness to receive monkeypox vaccination among MSM in Southern China, an area with a high incidence area of emerging infectious diseases. This present study aimed to investigate the level of monkeypox knowledge among the MSM population in Southern China and to understand their attitudes and willingness toward monkeypox vaccination. This research is crucial for assessing the effectiveness and implementing comprehensive interventions targeting this high-risk population.

## Methods

### Study participants

We a conducted cross-sectional survey on knowledge, worries, attitudes, behaviors and willingness toward monkeypox vaccination among MSM in Guangxi, Yunnan, and Hainan provinces of Southern China. The survey was administered online from August 22 to September 11, 2022. Participants were recruited through WeChat groups by trained leaders from local Social Organization for MSM population and staff from local CDC.

The eligibility criteria for the participants were as follows: (1) identify as male; (2) be 18 years of age or older; (3) self-identify as MSM; (4) have the ability to complete the questionnaire in Chinese online; and (5) provided online informed consent.

### Ethical considerations

The study was approved by the Human Research Ethics Committee of Guangxi Medical University (Aprroval number: 20220205). Respondents were recruited for participation and received a compensation of 10 RMB (about 1.43 USD). Prior to proceeding to the questionnaire page in the survey, participants were required to provide their informed consent.

### Sample size and technique

The sample size was calculated using the formula of the cross-sectional study. We conducted a pre-survey in Guangxi, and found that the rate of willingness to vaccinate against MPX was 70.6% (89/126).


$$n={\left(\frac{Z_{1-\alpha /2}}{\delta }\right)}^{2}\times p\times \left(1-p\right)$$

Here, = 0.05 (two sides), *Z* = 1.96, *δ* = 0.03, and the willingness rate to vaccinate against MPX (p) was 70.6%. Previous cross-sectional studies have reported that the non-response rates among MSM population in online surveys is ranging from 10 to 20% (31–33). Considering that we set an effective reward mechanism (34) that each participant who completed the online survey was rewarded with 10 RMB to reduce the non-response rate. Therefore, a non-response rate of 10% was set for this study, and determined a final sample size of 920.

### Survey measure

A Web-based, self-administered anonymous questionnaire was developed using the online survey platform Wenjuanxing.^1^ MSM participants accessed the questionnaire by clicking the web link or scanning the Quick Response (QR) code. The questionnaire consisted of the following sections: (1) Sociodemographic characteristics; (2) Sexual behavior characteristics; (3) Knowledge of the MPX and smallpox or monkeypox vaccines; and (4) Worries and attitudes about MPX and MPX vaccine; and (5) Willingness to receive MPX vaccination. Ultimately, 1,195 MSM completed the survey. After excluding 102 questionnaires with missing items or logically contradictory answers, a total of 1,093 valid questionnaires were included in the analysis.

### Sociodemographic characteristics

Sociodemographic characteristics were as follows: age, current residence area, marital status, education level, occupation, monthly income, history of chronic diseases, history of STDs, and HIV status. We also collected sexual behavior information, such as the number of sexual partners, the frequency of anal sex, and condom use with male sexual partners.

### Perceptions of MPX

This study assessed participants’ perceptions of monkeypox through a series of questions. In brief, participants were asked “Did you know about the MPXV before this survey?” (Yes/No). Participants who answered “Yes” were considered to be aware of MPX. For those who indicated awareness, further inquiries were made regarding the source of MPX knowledge, the sequelae of infection, the routes of transmission, and the symptoms associated with MPX. Similarly, participants were asked if they had ever heard of the smallpox (cowpox) vaccine, with response options of “Yes” or “No,” in order to assess their awareness of the monkeypox vaccine. Participants with perceptions of the monkeypox vaccine were then asked about their perceptions of the monkeypox vaccine’s safety, their knowledge of specific vaccines such as ACAM2000 (smallpox vaccine), and JYNNEOS (a monkeypox vaccine certified by the US Food and Drug Administration in 2019), the priority occupation for vaccination and the preventive effects of the monkeypox vaccine on HIV/acquired immunodeficiency syndrome (AIDS) patients.

### Knowledge of MPX

The participants’ knowledge of MPX was assessed using the following scale item questions, as follows: (1) People can be re-infected with the MPXV; (2) MPX cannot spread through aerosols; (3) Patients infected with the MPXV may experience residual sequelae; (4) The MPXV can be detected in semen; (5) There is little likelihood of gene mutation in the MPXV; (6) Most people infected with the MPXV will fully recover within a few weeks; (7) MSM is considered a high-risk group for MPXV infection; (8) The main mode of transmission for MPXV is through close contact; (9) People living with HIV account for a higher proportion of MPXV infections; (10) Clinical manifestations of MPX are similar between HIV-infected and HIV-uninfected individuals.

For each question, participants were provided with three response options: Disagree, Uncertain, and Agree. The response of “uncertain” indicated a lack of good knowledge regarding MPX. Good knowledge of MPX was defined as correctly answering six or more out of the 10 questions.

### Worries about MPX

Worries about MPX were assessed using the following 3 questions: (1) “Are you worried about the potential pandemic of monkeypox in mainland China?”; (2) “Are you worried about being infected with MPXV?”; and (3) “Are you worried about having sequelae after being infected with MPX?”; Each question provides participants with three response options Yes/No/I do not know. If participants answered “No” to question (1), they were further asked about the reasons for their belief that “MPX will/will not be pandemic in China.

### Attitudes toward monkeypox vaccine

Participants’ attitudes toward the monkeypox vaccine were asked by using the six items: (1) The smallpox/monkeypox vaccinee has adverse reactions; (2) The smallpox/monkeypox vaccine is not recommended for people living with HIV; (3) The smallpox vaccine has cross-protection effects against the MPXV; (4) The smallpox vaccine may have potential preventive effects against HIV infection; (5) The smallpox/monkeypox vaccine should be prioritized to MSM; (6) Pregnant women, people with cardiovascular conditions, those with skin disease are not recommended to receive the smallpox/monkeypox vaccine. Each question provides participants with three response options: Disagree/Uncertain/Agree. We also asked questions about the following questions regarding attitudes toward the MPX vaccine: (1) “Considering the monkeypox vaccine as safe,” (2) “Considering the monkeypox vaccine as more beneficial for people living with HIV,” (3) and “Being willing to receive monkeypox or smallpox vaccination when most of your friends or sexual partners have been. Vaccinated.”

### The willingness to received monkeypox vaccination

Participants’ willingness to receive vaccination was assessed using the following questions: “Do you think it is necessary to promote smallpox/monkeypox vaccination in China”; “Are you willing to encourage your friends or sexual partners to receive monkeypox vaccination when it becomes available and widely accessible?” These questions were used to assess the willingness toward monkeypox vaccination. Participants were also asked to provide reasons for their “willingness” or “unwillingness.” Additionally, we asked participants about their acceptable price range for smallpox/monkeypox vaccine.

### Questionnaire quality control

We conducted questionnaire quality control for the online survey as following: (1) A phone IP address only allowed participants to finish the questionnaire once to avoid repeating response. (2) There are two similar questions (a. Are you familiar with the monkeypox virus before participating in this survey? b. Do you know about monkeypox virus?) in a different section of the questionnaire. If the participant gives different response for these two similar questions, this record is considered invalid, and will be deleted from the analysis data. (3) We set one common-sense question (Among Shanghai, Nanjing, Beijing, and Wuhan, which city is the capital of China?) unrelated to the purpose of the study to eliminate the inattentive response. (4) A quality control researcher performed online check to ensure verify data quality. (5) The content validity was deemed acceptable, as Cronbach’s alpha was 0.892.

### Statistical analysis

Descriptive statistics were used to analyze the characteristics of the study population. Subsequently, frequencies were calculated. Pearson’s Chi-squared test was used to compare variables between participants’ willingness to vaccinate and their knowledge of and attitudes toward MPX. Multivariate logistic regression was used to explore the factors associated with willingness to adopt monkeypox vaccination. In the multivariate regression model, we included only single factors (chi-square test or *t*-test) that were statistically significant and professionally meaningful. The “Enter” method (*p* < 0.2) was employed to include all potentially influential factors as independent variables in the multivariable logistic regression model. Subsequently, adjusted odds ratios (aOR) and 95% confidence intervals (CI) were calculated. The Hosmer and Lemeshow test was used to assess the goodness of model fitting. Bar graphs were created using GraphPad Prism 8.0 (GraphPad Software, San Diego, CA) to illustrate participants’ responses to questions about MPX. All analyses were performed using SPSS25.0 (IBM Corporation, New York, NY, United States) and a two-sided *p*-value < 0.05 was considered statistically significant.

## Results

### Socio-demographic characteristics

**Figure 1:**
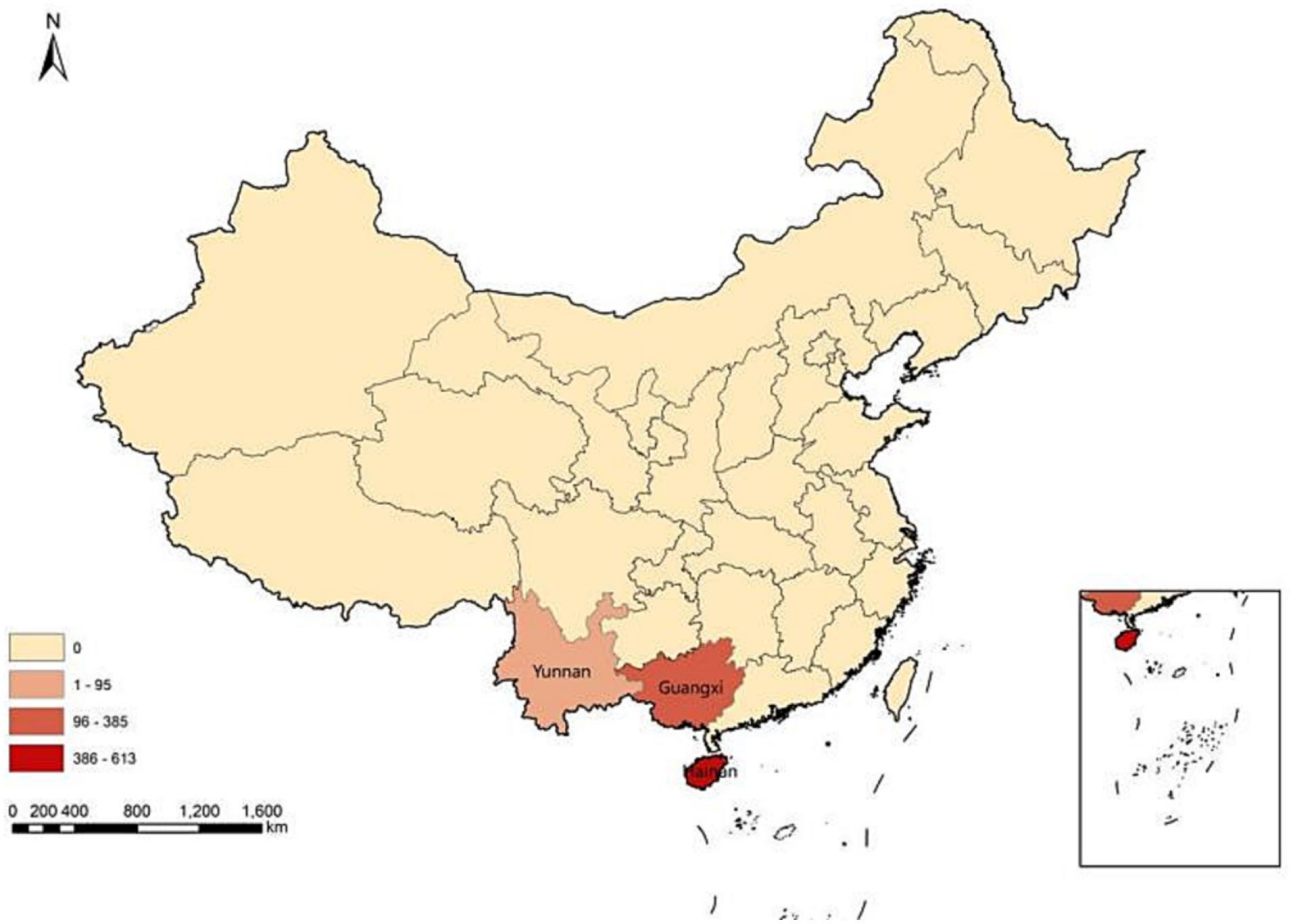
Geographical distribution of the participants.

A total of 1,093 participants were included in the study (Figure 1). As shown in Table 1, the participants had a mean age of 27.9 years (SD: 7.9, range: 18–70). A significant majority of the participants (91.5%) resided in urban areas. Among the participants, 78.2% were single, whereas 16.8% were married. In terms of education, approximately half of the participants (51%) had undergraduate education. Moreover, the majority of participants reported having no history of chronic diseases (78.9%) or sexually transmitted diseases (STDs) (78.6%). Additionally, 17.9% of the participants self-reported being HIV-infected. Other socio-demographic characteristics are shown in Table 1.

**Table 1:** Sociodemographic, knowledge, worries and attitudes toward MPX and MPX vaccine among MSM in China (*n* = 1,093).

Variables	Total (*N* = 1,093)	Unwilling (*n* = 94)	Willing (*n* = 999)	*P*-value
Sociodemographic				
Age, year				0.049
<26	518 (47.4)	41 (43.6)	477 (47.8)	
26–40	494 (45.2)	51 (54.3)	443 (44.3)	
>40	81 (7.4)	2 (2.1)	79 (7.9)	
Current residence area				0.439
Rural	93 (8.5)	10 (10.6)	83 (8.3)	
Urban	1,000 (91.5)	84 (89.4)	916 (91.7)	
HIV status				0.669
Negative	709 (64.9)	59 (62.8)	650 (65.1)	
Positive	196 (17.9)	20 (21.3)	176 (17.6)	
Unknown	188 (17.2)	15 (16)	173 (17.3)	
Marital status/social relationship status				0.001
Single (unmarried, divorced or widowed)	855 (78.2)	61 (64.9)	794 (79.5)	
Married	184 (16.8)	22 (23.4)	162 (16.2)	
Cohabitation	54 (5)	11 (11.7)	43 (4.3)	
Education				0.954
High school or below	181 (16.6)	15 (16)	166 (16.6)	
Junior or higher vocational college	260 (23.8)	24 (25.5)	236 (23.6)	
Undergraduate	557 (51)	46 (48.9)	511 (51.2)	
Postgraduate	95 (8.6)	9 (9.6)	86 (8.6)	
Occupation				0.486
Farmer or worker	120 (11)	14 (14.9)	106 (10.6)	
Student	227 (20.8)	14 (14.9)	213 (21.3)	
Company or government employee	455 (41.6)	39 (41.5)	416 (41.6)	
Business or self-employed	213 (19.5)	19 (20.2)	194 (19.4)	
Unemployed	78 (7.1)	8 (8.5)	70 (7)	
Monthly income				0.159
No fixed	211 (19.3)	18 (19.2)	193 (19.4)	
<2,000	98 (9)	8 (8.5)	90 (9)	
2,000-4,999	372 (34)	24 (25.5)	348 (34.8)	
5,000-9,999	297 (27.2)	28 (29.8)	269 (26.9)	
>10,000	115 (10.5)	16 (17)	99 (9.9)	
Sexual orientation				0.246
Heterosexual	402 (36.8)	37 (39.4)	365 (36.6)	
Homosexual	495 (45.2)	35 (37.2)	460 (46)	
Bisexual	155 (14.2)	16 (17)	139 (13.9)	
Pansexual and other	41 (3.8)	6 (6.4)	35 (3.5)	
History of chronic diseases				0.217
No	236 (21.6)	25 (26.6)	211 (21.1)	
Yes	857 (78.4)	69 (73.4)	788 (78.9)	
History of STDs				0.200
No	859 (78.6)	69 (73.4)	790 (79.1)	
Yes	234 (21.4)	25 (26.6)	209 (20.9)	
Knowledge of MPX and monkeypox vaccine				
Awareness of MPX				0.006
No	329 (30.1)	40 (42.6)	289 (28.9)	
Yes	764 (69.9)	54 (57.4)	710 (71.1)	
Frequency of following MPX information				0.953
Less than one times in a week	272 (35.6)	4 (7.4)	45 (6.4)	
One or more times in a week	443 (58)	19 (35.2)	253 (35.6)	
Not focused	49 (6.4)	31 (57.4)	412 (58)	
Having good knowledge of MPX				0.017
No	432 (39.5)	48 (51.1)	384 (38.4)	
Yes	661 (60.5)	46 (48.9)	615 (61.6)	
Being considered people could be re-infected MPXV				0.017
Disagree	76 (7)	12 (12.8)	64 (6.4)	
Unsure	347 (31.7)	35 (37.2)	312 (31.2)	
Agree	670 (61.3)	47 (50)	623 (62.4)	
Being considered MSM are high-risk population of MPXV infection				0.049
Disagree	91 (8.3)	11 (11.7)	80 (8)	
Uncertain	273 (25)	31 (33)	242 (24.2)	
Agree	729 (66.7)	52 (55.3)	677 (67.8)	
Having ever heard of smallpox (cowpox) vaccine				<0.001
No	139 (12.7)	27 (28.7)	112 (11.2)	
Yes	954 (87.3)	67 (71.3)	887 (88.8)	
Knowing a(Smallpox vaccine, ACAM2000) and b(JYNNEOS) are currently used to prevent MPX *				0.020
I do not know	460 (42.1)	52 (55.3)	408 (40.9)	
Having ever heard of a and b*	202 (18.5)	13 (13.8)	189 (18.9)	
Having ever heard of b*	81 (7.4)	9 (9.6)	72 (7.2)	
Having ever heard of a*	350 (32)	20 (21.3)	330 (33)	
Being considered monkeypox vaccine is better for people living with HIV				0.001
No	200 (18.3)	24 (25.6)	176 (17.7)	
Uncertain	416 (38.1)	46 (48.9)	370 (37)	
Yes	477 (43.6)	24 (25.5)	453 (45.3)	
Worries about MPX				
Worried about the potential pandemic in mainland China				0.007
No	292 (29.9)	22 (25.6)	270 (30.3)	
Yes	323 (33)	19 (22.1)	304 (34.1)	
I do not know	362 (37.1)	45 (52.3)	317 (35.6)	
Worried about being infected with MPXV				0.032
No	373 (34.1)	33 (35.1)	340 (34)	
Yes	364 (33.3)	21 (22.3)	343 (34.3)	
I do not know	356 (32.6)	40 (42.6)	316 (31.6)	
Worried about having sequelae after infected MPX				0.004
No	90 (8.2)	10 (10.6)	80 (8)	
Yes	639 (58.5)	40 (42.6)	599 (60)	
I do not know	364 (33.3)	44 (46.8)	320 (32)	
Attitudes about monkeypox vaccination				
Being considered monkeypox vaccine is safety				<0.001
No	95 (8.7)	16 (17)	79 (7.9)	
Yes	746 (68.2)	37 (39.4)	709 (71)	
Do not know	252 (23.1)	41 (43.6)	211 (21.1)	
Being considered the necessity for smallpox/monkeypox vaccination promotion in China				<0.001
No	64 (5.9)	32 (34)	32 (3.2)	
Yes	1,029 (94.1)	62 (66)	967 (96.8)	
Being considered smallpox/monkeypox vaccine should be prioritized for MSM				0.040
Disagree	107 (9.8)	14 (14.9)	93 (9.3)	
Unsure	325 (29.7)	34 (36.2)	291 (29.1)	
Agree	661 (60.5)	46 (48.9)	615 (61.6)	
Willingness to encourage male friends or sexual partners to get vaccinated against MPX				<0.001
Unwilling	60 (5.5)	33 (35.1)	27 (2.7)	
Willing	1,033 (94.5)	61 (64.9)	972 (97.3)	
Willingness to receive monkeypox or smallpox vaccination when most of your friends or sexual partners have been vaccinated				<0.001
Unwilling	49 (4.5)	31 (33)	18 (1.8)	
Willing	1,044 (95.5)	63 (67)	981 (98.2)	
Behaviors				
Number of anal sex partners in the past 3 months				<0.001
0	159 (24.1)	14 (26.4)	145 (23.9)	
1–5	454 (68.9)	28 (52.8)	426 (70.3)	
>6	46 (7)	11 (20.8)	35 (5.8)	
Frequency of oral sex in the past 3 months				0.005
3 times or below per month	343 (68.6)	18 (46.2)	325 (70.5)	
4–6 times per week	95 (19)	14 (35.9)	81 (17.6)	
4 times or above per week	62 (12.4)	7 (17.9)	55 (11.9)	
Frequency of condom use in the past 3 months				0.056
never	21 (4.2)	4 (10.3)	17 (3.7)	
1–39%	23 (4.6)	4 (10.3)	19 (4.1)	
40–60%	52 (10.4)	6 (15.4)	46 (10)	
61–99%	125 (25)	7 (17.9)	118 (25.6)	
100%	279 (55.8)	18 (46.2)	261 (56.6)	
Having ever uptake of smallpox vaccine at birth				0.001
No	247 (22.6)	34 (36.2)	213 (21.3)	
Yes	846 (77.4)	60 (63.8)	786 (78.7)	
Having ever uptake monkeypox/smallpox vaccine in the last year				0.776
No	718 (65.7)	63 (67)	655 (65.6)	
Yes	375 (34.3)	31 (33)	344 (34.4)	

*a, Smallpox vaccine (ACAM2000); b, JYNNEOS, a monkeypox vaccine certified by the US Medicine and Drug Administration in 2019. MSM, men who have sex with men; HIV, human immunodeficiency virus; STDs, Sexually transmitted diseases.

### Knowledge about MPX and monkeypox vaccine

According to Table 1, 69.9% of the participants were aware of MPX, and among them, 60.5% had good knowledge about MPX. Additionally, only 6.4% of participants self-reported not focusing on MPX information.

In addition, regarding the possibility of reinfection with MPXV, a majority of participants (61.3%) agreed that it is possible. Among these participants, 62.4% expressed willingness to receive the monkeypox vaccine. Furthermore, 66.7% of the participants recognized MSM as a high-risk group for MPXV infection, and among them, 67.8% expressed willingness to receive the monkeypox vaccine. Additionally, 43.6% of the participants agreed that the monkeypox vaccine is beneficial for individuals living with HIV (Table 1).

In terms of awareness of the smallpox (vaccinia) vaccine, a significant majority of participants (87.3%) reported being familiar with it. Among those who were aware, 88.8% expressed willingness to receive the monkeypox vaccine. In contrast, 42.1% of participants were unaware of the currently available vaccine against MPXV, and a majority of them (55.3%) expressed unwillingness to be vaccinated (Table 1). Meanwhile, The main reason for unwillingness is that they were concerned about serious adverse side effects (52.1%) Figure 4B).

**Figure 2:**
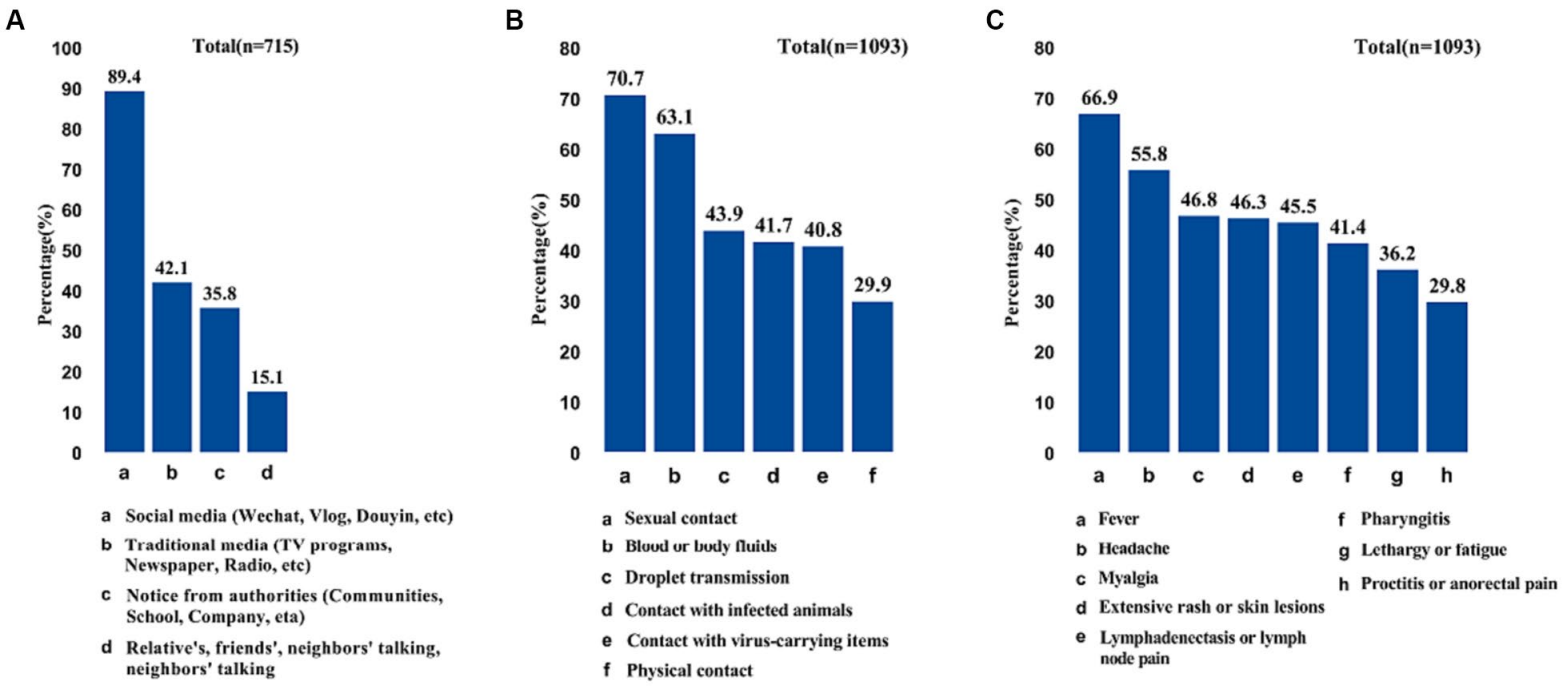
Sources of monkeypox information **(A),** dominant transmission route of monkeypox **(B)**, and participates’ perceived symptoms of monkeypox **(C)**.

It is shown that the majority of participants (89.4%) acquired monkeypox knowledge through social media (Figure 2A). A high proportion of participants considered sexual contact (70.7%) and contact with blood or body fluids (63.1%) as the primary modes of MPX transmission, followed by droplet transmission (43.9%) and contact with infected animals (41.7%) (Figure 2B). Regarding the symptoms of monkypox, most participants identified fever (66.9%), headache (55.8%), and myalgia (46.8%) as the main symptoms of MPX infection (Figure 2C).

**Figure 3:**
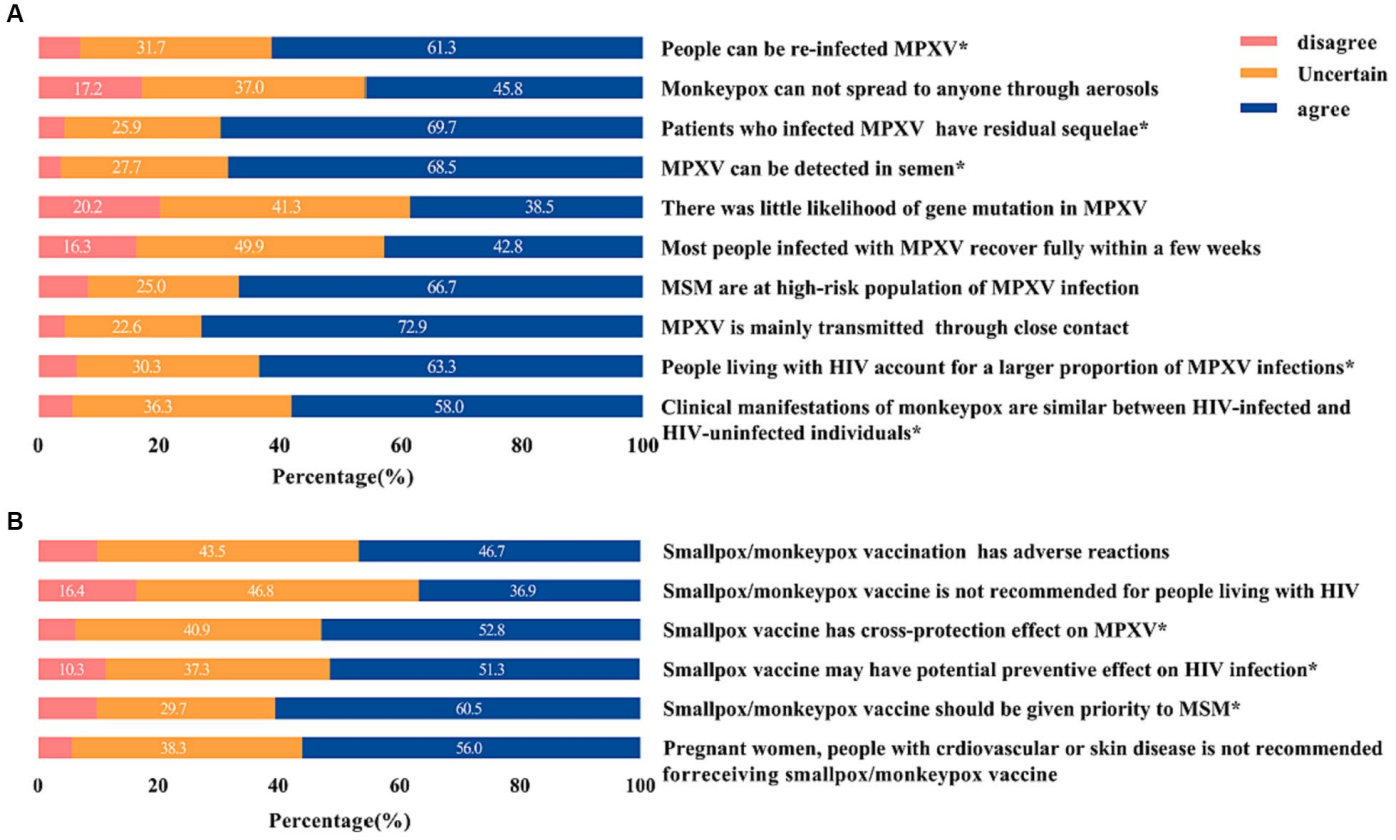
Detail response of knowledge about monkeypox **(A)** and monkeypox vaccine **(B)** among MSM in China.

**Figure 4:**
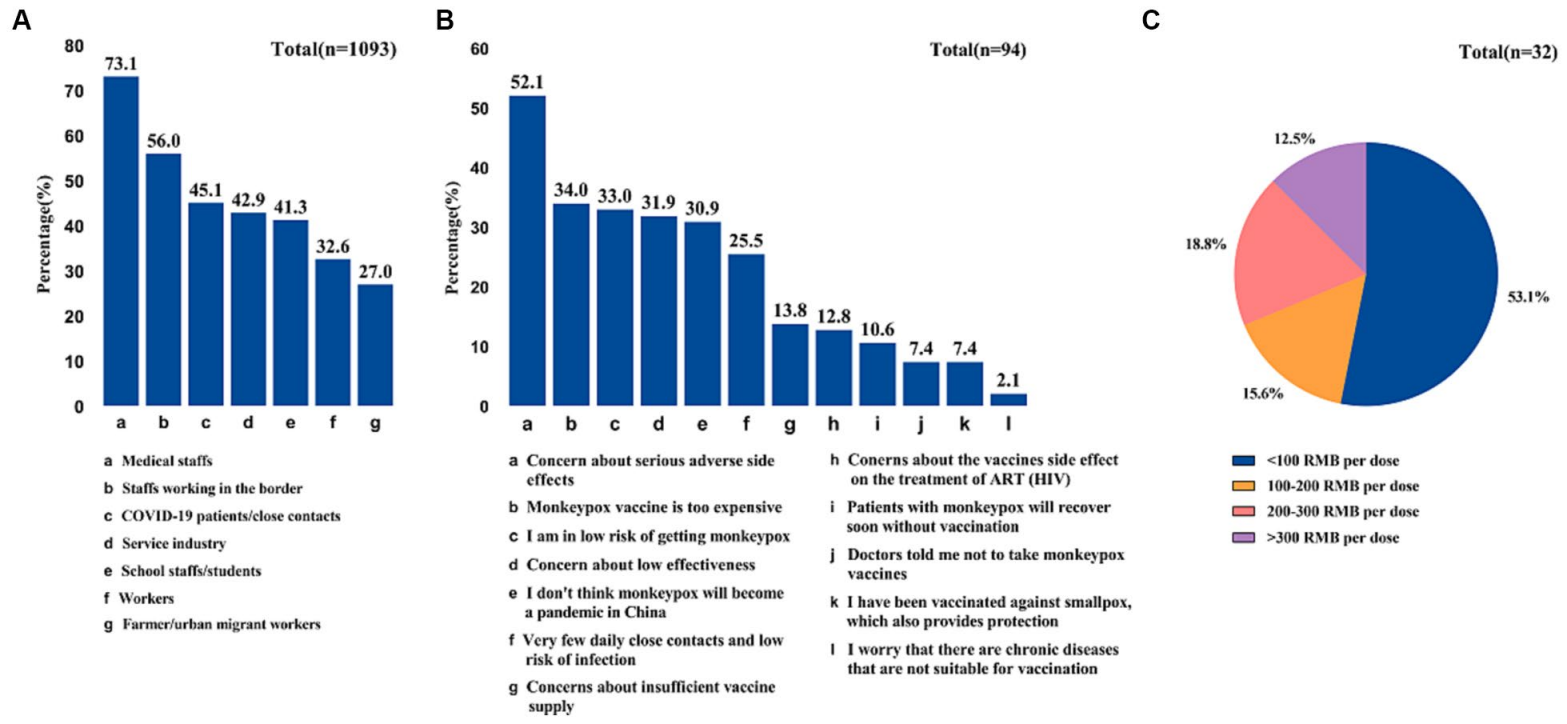
Participates’ perceived priority occupations for monkeypox vaccination **(A)**, reasons of unwillingness to vaccinate against monkeypox **(B)**, and expensive price range of smallpox/Monkeypox vaccine **(C)**.

Notably, almost half of MSM were unfamiliar with any new monkeypox vaccine (42.1%), such as ACAM2000 and JYNNEOS, and 60.5% of MSM believed that the vaccine should be prioritized for themselves (Figure 3B). In terms of vaccination priority based on occupation, participants identified medical staff (73.1%) and border workers (56%) as the highest priority groups for vaccination (Figure 4A).

Over 50% of participants provide accurate responses to each question. Detailed responses regarding knowledge about monkeypox and the monkeypox vaccine were shown in Figure 3.

### Worries about MPX

According to Table 1, approximately one-third of the people (33%) were worried about the potential pandemic in mainland China, while a similar proportion (33.3%) expressed concerns about being infected by MPXV. Furthermore, 58.5% of the participants were worried about the sequelae of MPX infection.

**Figure 5:**
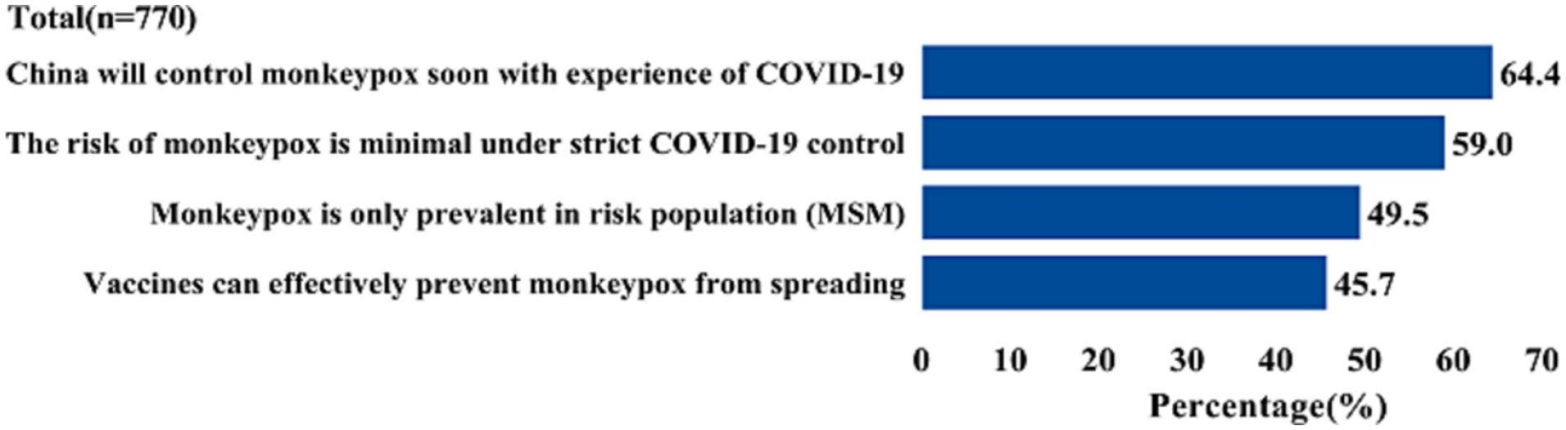
Reasons of monkeypox will not be a pandemic in China.

Figure 5 shows that 29.9% of the participants were not concerned about the monkeypox epidemic in China for the following reasons: (1) 64.4% of the participants believed that, based on the experience of COVID-19 prevention and control, the Chinese government could rapidly respond to the monkeypox epidemic; and (2) 59% of participants believed that the government’s strict COVID-19 control measures would effectively manage the MPX endemic in China.

### Attitudes about monkeypox vaccination

Table 1 reveals the attitudes regarding MPX vaccination. Of the participants, 68.2% considered the MPX vaccine to be safe, and 94.1% expressed support for its promotion in China. The majority (95.5%) expressed willingness to receive the vaccine themselves and 94.5% were willing to encourage their male friends or sexual partners to get vaccinated for monkeypox.

Furthermore, 34% of the participants expressed concerns about the high cost of the vaccine, considering prices exceeding 100 RMB/dose as expensive (Figure 4C).

### Behaviors

Among the participants, 68.6% reported engaging in oral sex three times or less per month, and a significant majority (70.5%) of them expressed willingness to receive the monkeypox vaccine. It is noteworthy that a majority (77.4%) of the participants had received smallpox vaccination at birth, and despite this, 78.7% still showed willingness to be vaccinated against MPX.

Table 1 Sociodemographic, knowledge, worries and attitudes toward MPX and MPX vaccine among MSM in China (*n* = 1,093).

### Correlates of willingness to receive monkeypox vaccination

Table 2 presented the correlates of willingness to adopt monkeypox vaccination among MSM. A significant majority of participants (94.1%, *N* = 1,029) expressed willingness to adopt monkeypox vaccination during the current epidemic. Factors associated with a higher likelihood of willingness to receive the vaccine included perceiving the vaccine as safe (aOR = 4.82, 95% CI: 1.35–17.18), agreeing on the necessity of government promotion of smallpox/monkeypox vaccination in China (aOR = 6.03, 95% CI: 1.07–33.93), believing that the smallpox/monkeypox vaccine should be prioritized for MSM (aOR = 5.01, 95% CI: 1.10–22.71) and having friends or sexual partners who had already received the monkeypox vaccination (aOR = 10.37, 95% CI: 2.11–50.99). Conversely, being married (aOR = 0.13, 95% CI: 0.03–0.47), engaging in anal sex 4–6 times per week in the past 3 months (aOR = 0.26, 95% CI: 0.09–0.77) were associated with a lower likelihood of accepting the monkeypox vaccination.

**Table 2:** Correlates of willingness to receive monkeypox vaccination.

Variables	aOR (95% CI)	*P*-value
Sociodemographic		
Age, year		
<26	Ref	
26–40	0.90 (0.34–2.38)	0.830
>40	5.31 (0.30–92.53)	0.252
HIV status		
Negative	Ref	
Positive	0.79 (0.21–2.91)	0.720
Unknown	4.10 (0.40–41.92)	0.235
Marital status/social relationship status		
Single (unmarried, divorced or widowed)	Ref	
Married	0.13 (0.03–0.47)	0.002
Cohabitation	0.38 (0.09–1.71)	0.208
Monthly income		
No fixed	Ref	
<2,000	0.41 (0.07–2.44)	0.324
2,000-4,999	2.31 (0.49–10.79)	0.289
5,000-9,999	0.71 (0.16–3.18)	0.657
>10,000	0.93 (0.13–6.76)	0.942
History of STDs		
No	Ref	
Yes	0.84 (0.25–2.80)	0.771
Knowledge of MPX and monkeypox vaccine		
Awareness of MPX		
No	Ref	
Yes	0.55 (0.17–1.76)	0.309
Having good knowledge of MPX		
No	Ref	
Yes	2.72 (0.67–11.09)	0.162
Being considered one person could be re-infected with MPXV		
Disagree	Ref	
Unsure	1.61 (0.22–11.74)	0.640
Agree	0.58 (0.09–3.85)	0.574
Being considered MSM are at high-risk population of MPXV infection		
Disagree	Ref	
Uncertain	0.37 (0.07–2.14)	0.268
Agree	0.60 (0.10–3.81)	0.590
Having ever heard of smallpox (cowpox) vaccine		
No	Ref	
Yes	1.48 (0.34–6.46)	0.604
Worries about MPX		
Worried about MPX epidemic in mainland China		
Impossible	Ref	
Possible	1.71 (0.46–6.42)	0.428
I do not know	0.65 (0.18–2.41)	0.521
Worried about being infected with MPXV		
Impossible	Ref	
Possible	0.93 (0.27–3.16)	0.903
I do not know	0.62 (0.18–2.10)	0.438
Worried about having sequelae after infected MPX		
Impossible	Ref	
Possible	1.01 (0.15–7.05)	0.991
I do not know	2.56 (0.25–25.92)	0.425
Attitudes about monkeypox vaccination		
Being considered monkeypox vaccine is safety		
No	Ref	
Yes	4.82 (1.35–17.18)	0.015
Do not know	1.26 (0.29–5.49)	0.761
Being considered of the necessity for promotion of smallpox/monkeypox vaccination in China		
No	Ref	
Yes	6.03 (1.07–33.93)	0.041
Being considered smallpox/monkeypox vaccine should be prioritized for MSM		
Disagree	Ref	
Unsure	1.61 (0.37–7.10)	0.529
Agree	5.01 (1.10–22.71)	0.037
Willing to encourage male friends or sexual partners to receive monkeypox vaccination when it was produced and nationally generalizable		
Unwilling	Ref	
Willing	3.19 (0.68–15.00)	0.142
Willingness to receive monkeypox or smallpox vaccination in the context of friends’ or sexual partners’ vaccination Status		
Unwilling	Ref	
Willing	10.37 (2.11–50.99)	0.004
Behaviors		
Frequency of anal sex in the past 3 months		
3 times or below per month	Ref	
4–6 times per week	0.26 (0.09–0.77)	0.015
4 times or above per week	0.50 (0.12–1.99)	0.322
Having ever uptake of smallpox vaccination at birth		
No	Ref	
Yes	1.57 (0.50–4.92)	0.443

CI: confidence interval; HIV: human immunodeficiency virus; MSM: men who have sex with men; MPXV: monkeypox virus; STDs: Sexually transmitted diseases; Ref: reference.

## Discussion

This study revealed that a noteworthy proportion (60.5%) of MSM demonstrated good knowledge of MPX. Additionally, 33% of the participants expressed concern regarding the potential pandemic of monkeypox in mainland China. Encouragingly, a substantial majority (91.4%) of the participants exhibited a positive attitude by expressing their willingness to receive the monkeypox vaccination. Several factors were found to be associated with this willingness, including marital status, lower frequency of recent anal sex, belief in the safety of the monkeypox vaccine, agreement with the promotion of smallpox/monkeypox vaccination, vaccination status of their friends or sexual partners, prior receipt of smallpox vaccination, and belief that priority should be given to MSM for the smallpox/monkeypox vaccine.

This survey revealed that a significant proportion of MSM demonstrated a high level of interest in and knowledge of MPX. It is noteworthy that despite only two reported cases in China before this survey (30), MSM exhibited a high level of interest in MPX. This could be attributed to their disproportionate representation in previous MPX outbreaks. The data from our study indicate that 60.5% of MSM exhibited good knowledge of MPX, which was higher than in other studies. For instance, the general population awareness of monkeypox infection stood at only 52% in previous research (35), medical doctors demonstrated good knowledge of MPXV in only 30.6% of cases (36), and only 49.4% of male sex workers exhibited good knowledge of MPX (27). This discrepancy may be attributed to variations in the study population, as well as differences in the items used to assess knowledge of MPX.

However, it is important to note that high interest in MPX and good knowledge of MPX cannot be directly interpreted as concern regarding a potential MPX pandemic. In this study, only one-third of participants expressed worry about the potential pandemic of MPX in mainland China. It is worth mentioning that the National Health Commission swiftly implemented stringent entry screening measures for both COVID-19 and MPX (37). Thus, a majority of participants (64.4%) believed that the Chinese government would effectively control monkeypox in the event of a pandemic, given their strict control measures for COVID-19. Although concerns about a potential pandemic were relatively low, nearly one-third of MSM expressed support for the promotion of monkeypox vaccination in China. This indicates a significant consensus among MSM regarding the importance of receiving the monkeypox vaccine, highlighting its potential as a powerful tool in preventing a potential epidemic (38).

This study revealed a higher willingness among MSM in southern China to receive the monkeypox vaccine. This finding is consistent with an earlier survey conducted in July 2022, which reported a 90.2% acceptance rate of the monkeypox vaccine among MSM (28). However, there were noticeable differences in vaccine willingness among different study populations, ranging from 56.8% (39) and 63% (27) among MSM to 68.8% in the general population (30). This study found a high willingness to receive the monkeypox vaccine, which could be attributed to other notable findings. Nearly two-thirds of the participants (64.4%) expressed significant concern about the recent MPX epidemic, and 33.3% of participants worried about being infected with MPXV. A similar study conducted in the Netherlands reported that 52% of MSM showed high concern about MPX, and 30% perceived themselves to be at high risk of infection (40). Additionally, the high willingness to vaccinate may be influenced by the increased risk of infection among MSM during the current MPX epidemic due to factors such as sexual contact (41–43). To enhance the promotion and education of the monkeypox vaccine among MSM in the HIV population, it is essential to disseminate accurate and comprehensive information about its safety, efficacy, and significance. Additionally, providing convenient vaccination services at easily accessible locations with flexible scheduling is crucial. Furthermore, targeted interventions should be implemented to address high-risk behaviors, including sexual contact, and to promote the uptake of the monkeypox vaccine among MSM.

The current study indicated that MSM are more likely to receive monkeypox or smallpox vaccination when most of their friends or sexual partners have already been vaccinated. The findings suggest that positive attitudes toward vaccination and frequent discussions about vaccinations with family and friends can influence individuals’ own attitudes toward vaccination, increasing the likelihood of getting vaccinated (44). Previous research has indicated that MSM may place importance on the viewpoints of their male sexual partners or peers (45). Therefore, it can be inferred that they are more likely to be willing to receive vaccinations when their friends or sexual partners are vaccinated, as it provides them with a sense of belonging. Social influence from family, friends, and peers can serve as a facilitating factor in promoting vaccine uptake through community education and advocacy programs. Another reason is that sex activities are believed to be a mode of transmission of MPX. MSM may engage in sexual activity with multiple partners or without protection, placing them at an increased risk of contracting MPX (46). Consequently, they perceive that the benefits of vaccination outweigh the risks (47). Moreover, MSM predominantly utilizes social media platforms to seek sexual partners and have extensive and interconnected social networks. Previous studies have also highlighted the role of social media in shaping vaccination decisions (48, 49). Therefore, it is crucial to prioritize vaccination efforts and engage individuals who are similar to the MSM population. Utilizing social media can be instrumental in enhancing the acceptance of the monkeypox vaccine among the MSM population.

As observed in this study, MSM who considered the vaccine to be safe exhibited a high willingness to receive monkeypox vaccination. Trust in the safety and effectiveness of vaccines plays an important role in an individual’s decision to receive vaccination (50). The safety of Chinese vaccines (51, 52) has gained widespread recognition among the general population, which increases the trust of MSM in the vaccine. Among participants who were unwilling to receive the vaccine, half of them (52.1%) cited concerns about the safety and side effects of the vaccine as the main reason for their unwillingness. Hence, confidence in the safety and effectiveness of the vaccine constitutes a crucial factor for individuals who are hesitant to undergo vaccination.

The participants in this research expressed a preference for the price of monkeypox vaccine to be set at the lowest tier, which is less than 100 RMB per dose ($14.7 per dose). A cross-sectional study conducted in Indonesia revealed that the high personal financial cost of vaccination reduced its acceptability. Participants preferred free vaccinations or vaccinations priced at $37, or partially or fully subsidized by the government at $17.9, if higher vaccination coverage was needed (53). Another study (54) considered $7.5 as the lowest average cost per citizen. In comparison, the free vaccination policy implemented by the Chinese government (55) appears to be more conducive to increasing vaccination coverage and making it more convenient for the MSM population to receive vaccinations. This may potentially increase the willingness to receive monkeypox vaccination among MSM.

MSM who agreed that priority should be given to the high-risk population when vaccines are insufficiently available were more likely to receive vaccinations. This preference can be attributed to their perception of being at a high risk of MPX infection, which increases their inclination toward vaccination as a preventive measure. Furthermore, social norms and peer influence may also impact vaccine decision-making (45, 56). Therefore, it is imperative to provide monkeypox or smallpox vaccinations to MSM who are at a higher risk of monkeypox exposure in order to aid in controlling the recent outbreak of the virus.

Our findings revealed that participants who engaged in annual intercourse 4–6 times per week over the past 3 months were less likely to be vaccinated, in contrast to the findings of Fu et al. (39) and Ibuka et al. (57). It is possible that the sample size of participants who reported engaging in anal sex 4–6 times per week was small. Compared with single participants, married MSM were less likely to be vaccinated. The possible explanation could be that married individuals tend to be old, specifically in the age group of 41–50 years (58). Younger MSM, on the other hand, may have greater familiar with the internet and have better access to the latest information of emerging infectious disease (27). This facilitates their acquisition of accurate knowledge about MPX and improves their acceptance of vaccines, thus aiding in the promotion of vaccination among the general public. Moreover, singles with more sexual partners correlate with increased transmission risk and align with a greater willingness for vaccination than married MSM (59).

Some limitations of this study should be acknowledged. Firstly, it is important to note that the majority of participants in this survey were from urban areas and had an undergraduate education or above. This may impact the representativeness and generalizability of the study findings. Secondly, the assessment of willingness to vaccinate was based on a single question, which provided simplicity and efficiency but may lack multidimensionality. Therefore, future studies should consider adopting more comprehensive measures to assess willingness to vaccinate. Thirdly, due to the cross-sectional design of the study, causal inferences cannot be drawn. Fourthly, the self-designed questionnaire used in this study included self-reported and sensitive sexual questions, which could introduce reporting bias or recall bias. Lastly, it is important to note that the online questionnaire was only distributed through certain social organizations in three provinces in South China. This may result in a limited number of participants from this specific area, thereby limiting the generalizability of the study findings to other regions or populations.

## Conclusion

Despite these limitations, our study found a high willingness to vaccinate against monkeypox. Hesitancy to vaccinate against MPX was low, but it may still influence the scale-up of vaccination in China. Therefore, it is crucial to implement effective and comprehensive efforts to improve awareness and acceptance of the monkeypox vaccine among MSM. Specifically, attention should be given to MSM who engage in high-frequency anal sex, are married, express concerns about vaccine safety, and believe vaccination is unnecessary. Additionally, tailored vaccination campaigns targeting MSM should actively involve their peers.

## Data availability statement

The original contributions presented in the study are included in the article/supplementary material, further inquiries can be directed to the corresponding authors.

## Ethics statement

The study was approved by the Human Research Ethics Committee of Guangxi Medical University (Approval number: 20220205). Respondents were recruited for participation and received a compensation of 10 RMB (about 1.43 USD). Prior to proceeding to the questionnaire page in the survey, participants were required to provide their informed consent.

## Author contributions

X-JH: Writing – original draft, Writing – review & editing. Z-FL: Writing – original draft, Writing – review & editing. JQ: Investigation, Project administration, Supervision, Validation, Writing – review & editing. D-EY: Investigation, Writing – review & editing. FZ: Investigation, Project administration, Supervision, Validation, Writing – review & editing. G-GF: Investigation, Data curation, Formal analysis, Methodology, Project administration, Writing – review & editing. XC: Data curation, Formal analysis, Investigation, Methodology, Project administration, Writing – review & editing. J-FH: Data curation, Formal analysis, Investigation, Methodology, Project administration, Writing – review & editing. PC: Data curation, Formal analysis, Investigation, Methodology, Project administration, Writing – review & editing. ML: Investigation, Project administration, Supervision, Validation, Writing – review & editing. R-JZ: Investigation, Project administration, Supervision, Validation, Writing – review & editing. TL: Project administration, Software, Supervision, Validation, Writing – review & editing. J-JJ: Writing – original draft, Writing – review & editing. S-QA: Writing – original draft, Writing – review & editing. HL: Writing – original draft, Writing – review & editing. LY: Writing – original draft, Writing – review & editing. B-YL: Writing – original draft, Writing – review & editing.

## Footnotes

^1^https://www.wjx.cn
